# Effectiveness and feasibility of long-lasting insecticide-treated curtains and water container covers for dengue vector control in Colombia: a cluster randomised trial

**DOI:** 10.1093/trstmh/tru208

**Published:** 2015-01-19

**Authors:** Juliana Quintero, Tatiana García-Betancourt, Sebastian Cortés, Diana García, Lucas Alcalá, Catalina González-Uribe, Helena Brochero, Gabriel Carrasquilla

**Affiliations:** a Centro de Estudios e Investigación en Salud, CEIS-Fundación Santa Fe de Bogotá, Bogotá, Colombia; b Universidad Nacional de Colombia, Bogotá, Colombia

**Keywords:** *Aedes*, Colombia, Dengue, Long-lasting insecticide-treated nets, Public health, Randomised controlled trial

## Abstract

**Background:**

Long-lasting insecticide-treated net (LLIN) window and door curtains alone or in combination with LLIN water container covers were analysed regarding effectiveness in reducing dengue vector density, and feasibility of the intervention.

**Methods:**

A cluster randomised trial was conducted in an urban area of Colombia comparing 10 randomly selected control and 10 intervention clusters. In control clusters, routine vector control activities were performed. The intervention delivered first, LLIN curtains (from July to August 2013) and secondly, water container covers (from October to March 2014). Cross-sectional entomological surveys were carried out at baseline (February 2013 to June 2013), 9 weeks after the first intervention (August to October 2013), and 4–6 weeks after the second intervention (March to April 2014).

**Results:**

Curtains were installed in 922 households and water container covers in 303 households. The Breteau index (BI) fell from 14 to 6 in the intervention group and from 8 to 5 in the control group. The additional intervention with LLIN covers for water containers showed a significant reduction in pupae per person index (PPI) (p=0.01). In the intervention group, the PPI index showed a clear decline of 71% compared with 25% in the control group. Costs were high but options for cost savings were identified.

**Conclusions:**

Short term impact evaluation indicates that the intervention package can reduce dengue vector density but sustained effect will depend on multiple factors.

## Introduction

In Colombia, around 26 million people (55% of the entire population) are at risk of contracting dengue.^1^ Transmission has increased over the past two decades and recent outbreaks in 2010 and 2013 had higher case numbers than in previous years.^2^ The estimated economic burden of dengue disease for Colombia is US$292 for ambulatory cases and US$1975 for severe dengue cases.^3^ Prevention and reduction of the burden of disease rest upon vector control. Chemical tools are also used to control the immature and adult stages of the vector in the form of insecticide-treated materials that have shown promising results in reducing household-level dengue vector densities,^4–6^ as well as other vectors. There are also non-chemical strategies that consist of environmental management^7^ and, recently, the release of transgenic vectors.^8^

However, the efficacy of these strategies is sub-optimal as a result of the reported limitations of vector control programmes.^9,10^

Pursuing the need of additional research to explore the potential efficacy of long-lasting insecticide-treated net (LLIN) in the context of constant demand of novel interventions for dengue programmes in Colombia, we report the short term results of the first cluster randomised controlled trial of LLINs against *Aedes aegypti* carried out in a dengue hyperendemic area of Colombia from July 2013 to May 2014. The study addressed the effectiveness and feasibility of LLINs deployed as curtains for doors and windows, and water container covers designed and implemented through community actions.

## Materials and methods

### Study site

The study was conducted in the urban area of Girardot (4°18' N, 74°48′ W), Colombia, located 120 km from Bogotá, the country's capital. With approximately 103 839 inhabitants^11^ and 20 845 houses, its main economic activity is tourism. Girardot has an annual mean temperature of 33.3°C, rainfall of 1220 mm and humidity of 66.3%, with bimodal rain seasons (March to May and October to November).^12^ Climate conditions, low altitude (400 metres above sea level) and urban concentration make the city one of the most dengue-endemic areas in Colombia.^1^

### Study design and sample size

The study followed a cluster randomised trial design with cross-sectional entomological surveys. A cluster was defined as a geographical area that included at least 100 private households, but also non-residential buildings and public spaces. A buffer zone of 100 metres between clusters was assured to prevent spillover effects.

The sample size was calculated as required for cluster randomised intervention studies^13^ using 10 clusters with 100 households for each study arm taking into account the following assumptions reported in previous studies:^4,14^ mean numbers of pupae per person pre- and post-intervention in treated clusters were assumed to be 3.0 and 0.3, respectively;^4^ negative binomial distribution considering an overdispersion of the dependent variable; dispersion coefficient of 0.02; and intra-cluster coefficient of 0.05. This reflected a desired power of 80% with significance level set at 5%.

### Selection, pairing and random allocation of clusters to study groups

**Figure 1.**
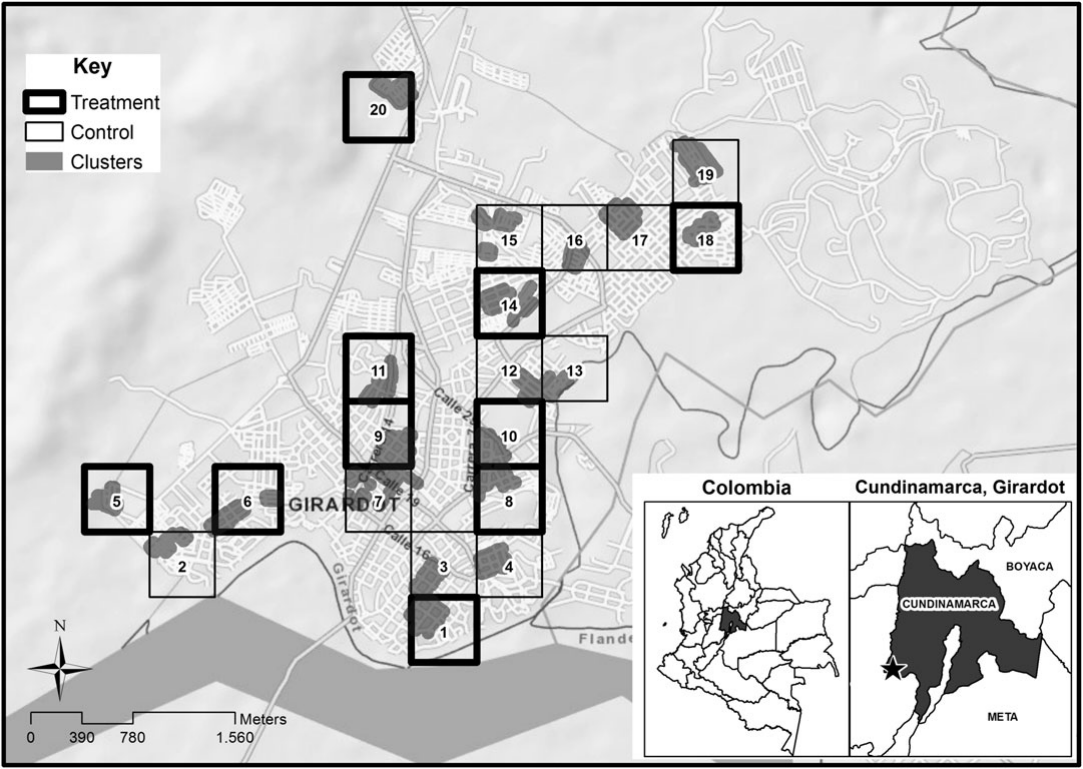
Map of study clusters of Girardot, Colombia, with cluster number and area. The figure shows selected clusters included in the study, they are listed from 1 to 20 for easy identification. Black squares represent intervened clusters with LLIN, grey ones represent control groups. The grey shade inside squares shows location of 100 households corresponding to each cluster.

To obtain a sample of 20 clusters, a map of Girardot was overlaid with a grid containing 200 squares as described by Quintero et al.^15^ Simple random numbers from Epi Info™ 2000 software (CDC, Atlanta, GA, USA) were used to select 20 squares, from the total of 196 identified (Figure 1).

Before random allocation of clusters to study groups (10 to control and 10 to intervention) a two-stage cluster analysis was used to define homogeneity between clusters regarding the following variables: entomological indices in wet and dry seasons, cluster socio-economic stratum, pupae per person index (PPI), Breteau index (BI), number of containers per household, size of cluster (in hectares) and inhabitants per cluster. Two groups of clusters were defined: one of 16 and the other of 4. Clusters were randomly allocated within the two groups based on a public raffle. Half (8) of the 16 clusters in one stratum and half (2) in the other were randomly distributed by raffle in the two study arms.

### Interventions

Situational analysis of the first phase of the project^15^ showed that low concrete water containers (‘albercas’) located in the backyards of houses were the most permanent productive container type for vector development. Stored water is used for washing, house cleaning and personal hygiene, and water shortages and cost savings are the main reasons for storage (García-Betancourt T, Higuera D, González-Uribe C et al., manuscript submitted).

PermaNet 2.0^®^ (long-lasting insecticide net treated with deltamethrin 50 mg/m^2^, Vestergaard-Frandsen, Lausanne, Switzerland) used as curtains on windows and doors as well as LLIN covers for household water container covers were selected as suitable intervention tools based on literature;^4–6,16,17^ natural populations of *Aedes aegypti* in Girardot being susceptible to pyrethroids,^18^ an insecticide authorised by public health authorities; and water containers (albercas or plastic containers of >200 L)^10,19^ with no protection against mosquito oviposture being the main productive sources of *Aedes aegypti* pupae (70% of all pupae were found in these containers) (Alcalá L, Quintero J, González-Uribe C et al., manuscript submitted). In addition, involvement of the community in all stages of the intervention encouraged acceptance of the project^20^ and represented an income-generating activity for local people.

**Figure 2.**
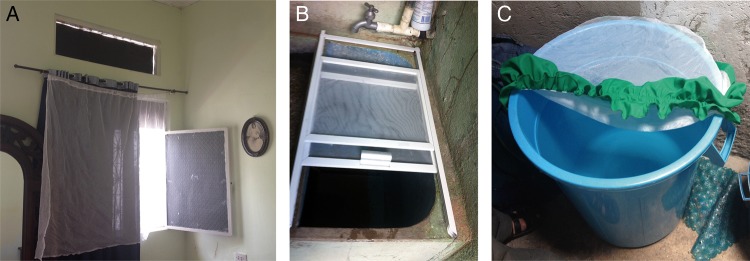
Curtains in windows, rectangular container cover and circular container cover. The Figure shows three intervention tools. (A) a curtain inside a household in Girardot. (B) the rectangular water container cover for rectangular or square cement containers of at least 200 L capacity; the cover is made of an aluminum frame, LLIN netting and a sliding mechanism for opening both doors. (C) the circular cover made of rubric, LLIN netting and rubber, for plastic and cement cylindrical containers that can store more than 200 L. This figure is available in black and white in print and in colour at Transactions online.

The interventions were applied at the household level (Table 1). Houses received: curtains for windows and doors: 2852 curtains (about three per household) and 947 door curtains (one per house) were sewn by 10 seamstress from white bed-nets of PermaNet 2.0, WHOPES approved^21^ (Figure 2A) and covers for water containers of >200 L (Figures 2B and 2C). Two types of water containers were designed through workshops within the community as described in Garcia-Betancourt T et al.^22^ and manufactured by 4 microenterprises and 10 seamstress. The design depended on the dimensions and shapes of the water containers. For cylindrical tanks, PermaNet 2.0 circular lids were made by seamstress with an elastic band and waterproof material for fixing. Square and rectangular covers consisted of aluminium frames with a sliding mechanism fixed to PermaNet 2.0 and were made and installed by small to medium enterprises (SMEs).

**Table 1.** Number of curtains and water container covers installed per cluster until May 2014, Girardot, Colombia

Cluster	Accepted interventions	Curtains											Covers			
		Number of curtains installed per cluster				Number of curtains installed per household							Houses^b^	Covers^b^	Low water containers	
		Houses	Curtains	Window curtains installed	Door curtains installed	Windows^a^	Mean windows per cluster	Doors	Mean doors per cluster	Mean of curtains installed	Mean of window curtains installed	Mean of door curtains installed			Per cluster	Per house
1	100	98	340	242	98	341	3.5	493	5.0	3.5	2.5	1	NA	NA	94	0.94
5	96	98	289	191	98	175	1.8	325	3.3	2.9	1.9	1	80	118	136	1.42
6	100	97	398	301	97	324	3.3	446	4.6	4.1	3.1	1	NA	NA	89	0.89
8	95	94	366	272	94	325	3.5	557	5.9	3.9	2.9	1	NA	NA	85	0.89
9	74	67	232	165	67	312	4.7	432	6.4	3.5	2.5	1	NA	NA	61	0.82
10	99	97	339	242	97	285	2.9	476	4.9	3.5	2.5	1	NA	NA	74	0.75
11	96	86	275	189	86	333	3.9	437	5.1	3.2	2.2	1	77	90	97	1.01
14	94	93	277	184	93	312	3.4	391	4.2	3.0	2.0	1	NA	NA	66	0.70
18	98	97	333	236	97	315	3.2	357	3.7	3.4	2.4	1	79	79	101	1.03
20	95	95	289	194	95	254	2.7	255	2.7	3.0	2.0	1	67	67	67	0.71
Total	947	922	3138	2216	922	2976	3.2	4169	4.5	3.4	2.4	1	303	354	870	9.16

NA: not applicable.

^a^ Total number of existing windows.

^b^ Installation in progress in clusters 1, 6, 8, 9, 10 and 14.

First, curtains were installed immediately after baseline entomological surveys by the community in 922 intervention houses from July to August 2013. They were fixed by homeowners with a white string and two nails beneath existing curtains (in some homes) and usually supervised by research staff. At least one door per house and all unprotected and in-use windows were covered. However, it was not possible to cover the openings above windows if present (Figure 2A).

The second intervention, water container covers, were implemented in the same houses that were previously allocated to curtains, 8 months after baseline surveys. A total of 354 lids (1.2 per household) in 4 clusters representing 303 households out of 385 have been installed so far from October to March 2014.

In addition, during 2013 the vector control programme continued to deliver the routine interventions regarding larvae control through larvicides (Abate, American Cyanamid Co., Princeton, NJ, USA), health education, and occasional public space spraying of an ultra-low volume of Malathion (Southern Agricultural Insecticides Inc., Palmetto, FL, USA) in both control and treatment clusters.

### Data collection

#### Entomological data: pupal and larval surveys

Cross-sectional entomological surveys were conducted in public and private premises by vector control technicians and biologists from the research team following standard operational procedures.^23^ All accessible water containers in intervention and control clusters were inspected. All pupae in breeding sites were collected and examined for species and sex in a laboratory.

The surveys were conducted at baseline over a period of 4 months (February 2013 to June 2013—dry and wet seasons, respectively), 9 weeks after first intervention (August to October 2013), and 4 to 6 weeks after the second intervention (March to April 2014).

#### Coverage, use and satisfaction assessment

For assessing the community coverage, use and satisfaction of the intervention, a semi-structured questionnaire was developed about people's willingness to pay, perceived effectiveness and quality, adverse events, curtains covering windows and material conditions (damage, cleanness). Household heads were interviewed by field staff supervised by research team members. In addition, 18 semi-structured interviews and 14 focal group discussions (FGDs) were organised with local people by an anthropologist (10 evaluating curtains and 4 evaluating container covers).

#### Cost analysis

Using a micro-costing approach data on quantities of resources used and unit costs were collected according to the resources consumed, recurrent costs (personnel, consumables, operating costs) and capital costs (vehicles, equipment). No overhead costs were included. Comparable information was requested from the routine vector control programmes to estimate incremental costs.

### Analysis

#### Data management

All data were double checked by field supervisors before being entered for quality assurance into a spreadsheet by trained personnel. The analysis was conducted in Stata version 13 (StataCorp LP, College station, TX, USA).

Our primary end point, PPI, defined by Focks^24^ and secondary outcomes BI and container index (CI) were calculated at cluster level. To adjust for cluster random effects a mixed model was used (xtmixed command).

**Figure 3.**
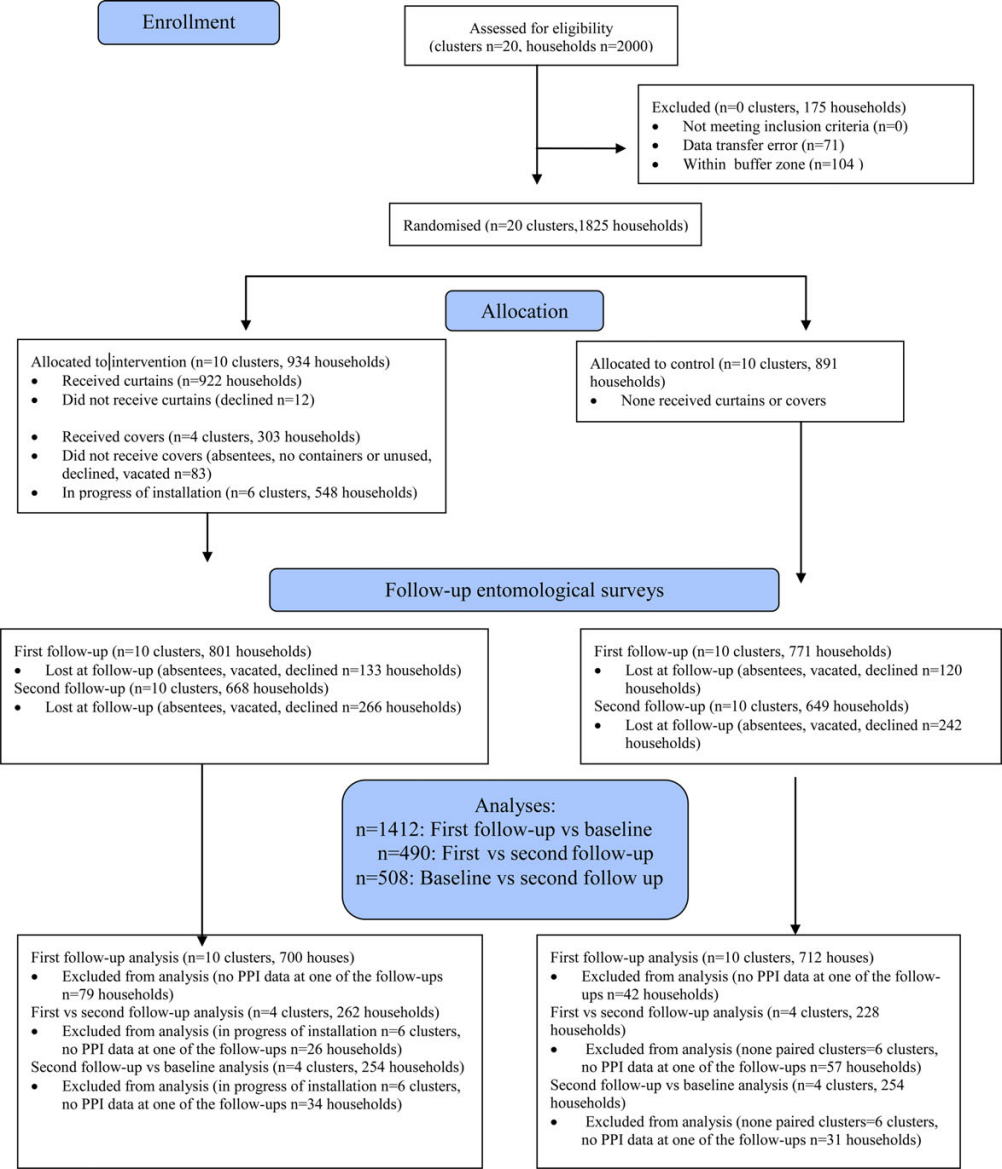
Flow diagram of households in Girardot through the study. A cluster randomised design with a sample of ten clusters per arm was used. All households were eligible. Randomisation was performed between arms. The figure represents the flow of clusters and households through the study. Six clusters without implemented covers were excluded from the analysis. This figure is available in black and white in print and in colour at Transactions online.

To estimate the reduction in vector densities between the study groups the difference-in-differences method was used.^25^ The significance of the differences was calculated using a non-parametric Z-test (ranksum command), as the number of observations in the data set does not satisfy parametric assumptions. In addition a t student test, weighted by cluster size was used to estimate CIs of the effect. Differences between three points were considered for the estimation: between baseline and first follow-up (curtains); between baseline and second follow-up (curtains and container covers); and between first and second follow-ups (see Figure 3). For the first follow-up 20 clusters were included in the analysis. For the second follow-up only eight clusters were included, four clusters where the second intervention was completed were chosen and compared to four control clusters within the same geographic area (See Figure 3).

A descriptive analysis regarding satisfaction, use and coverage was conducted. Costs were descriptively analysed and aggregated to calculate total costs, costs per house and incremental costs of the intervention over the routine vector control interventions (in terms of the cost per house).

## Results

All households were eligible: a total of 7162 people in 1825 households were included in the study of which 922 (50.5%) received curtains, 303 (16.6%) also received covers, and 891 (48.8%) were controls. Baseline data were collected from 1680 (92.1%) houses, first follow-up data for 1572 (86.1%) and 1317 (72.2%) for the second follow-up (Figure 3). For all follow-ups houses were revisited at least three times to maximise participation.

During the study period the total rainfall was 761 mm; the month with highest rainfall was May (22.4 mm) and the driest was July (9.2 mm). The majority of pupae collected from baseline surveys were *Aedes aegypti* from 189 containers of 5745 inspected. Therefore, all immature stages during follow-ups were assumed to be of this species. The majority of breeding sites were found to be indoors; the most common breeding sites were large water containers (2236, 38.9% of all breeding sites) and the most important in pupal production were albercas 1 and 2 which yielded 3006 pupae (69.5% of all pupae collected (n=4327) and low tanks that yielded 349 (8.1%). This trend remained relatively consistent after interventions were introduced and similar in both intervention and control clusters. Breeding sites like discarded and miscellaneous containers did not increase during follow-ups, which coincided with rainy seasons (Table 2).

**Table 2.** Breeding places and infestation level with immature *Aedes Aegypti* vector per study groups

	Intervention group						Control group					
	Baseline		Follow-up 1		Follow-up 2		Baseline		Follow-up 1		Follow-up 2	
Characteristics	Feb–Aug 2013 (wet–dry)		Oct–Dec 2013 (wet)		Mar–Apr 2014 (wet)		Feb–Aug 2013 (wet–dry)		Oct–Dec 2013 (wet)		Mar–Apr 2014 (wet)	
Precipitation	88.6		88.7		134		88.6		88.7		134	
Clusters	10	4	10	4	10	4	10	4	10	4	10	4
Households	843	347	801	327	668	288	837	324	771	282	649	284
Inhabitants	3231	1424	3157	1387	2696	1196	2975	1201	2898	1067	2415	1094
Containers	3253	905	3663	1340	2365	1462	2492	777	2761	878	1346	623
Low tanks (n)	10.1% (329)	18.2% (165)	4.3% (160)	8.4% (113)	12.3% (292)	10.3% (151)	9.7% (242)	10.2% (79)	3.0% (82)	5.6% (49)	16.1% (217)	17.0% (106)
Albercas (n)	25.9% (843)	35.5% (321)	19.5% (716)	21.6% (290)	26.3% (621)	18.8% (275)	33.0% (822)	38.5% (299)	23.2% (641)	26.2% (230)	43.0% (578)	37.6% (234)
Others (n)	64.0% (2081)	46.3% (419)	76.0% (2787)	70.0% (937)	61.4% (1452)	70.9% (1036)	57.2% (1428)	51.3% (399)	73.8% (2038)	68.2% (599)	40.9% (551)	45.4% (283)
Households with pupae	111	40	47	25	45	12	66	26	36	13	16	7
Containers with pupae	121	43	52	29	48	13	68	27	41	14	18	9
Pupaes	2932	1090	2929	1727	1022	276	1395	506	893	256	575	345
Pupae indices												
Container index	3.7%	4.8%	1.4%	2.2%	2.0%	0.9%	2.7%	3.5%	1.5%	1.6%	1.3%	1.4%
Household index	13.2%	11.5%	5.9%	7.6%	6.7%	4.2%	7.9%	8.0%	4.7%	4.6%	2.5%	2.5%
Breteau index	14.4	12.4	6.5	8.9	7.2	4.5	8.1	8.3	5.3	5.0	2.8	3.2
Pupae per person index	0.91	0.77	0.93	1.25	0.38	0.23	0.47	0.42	0.31	0.24	0.24	0.32
Pupae percentage per container type, relative contributions (n)												
Low tank	10.0 (293)	16.0 (174)	36.0 (1054)	54.0 (933)	1.0 (10)	0	4.0 (56)	12.0 (61)	2.0 (18)	4.0 (10)	3.0 (17)	6.0 (21)
Elevated tank	2.0 (59)	1.0 (11)	0	0	0	0	0	0	4.0 (36)	0	0	0
Alberca 1	41.0 (1202)	42.0 (458)	20.0 (586)	14.0 (242)	65.0 (664)	100 (100)	50.0 (698)	42.0 (213)	28.0 (250)	30.0 (77)	69.0 (397)	91.0 (314)
Alberca 2	23.0 (674)	31.0 (338)	39.0 (1142)	29.0 (501)	28.0 (286)	0	31.0 (432)	24.0 (121)	32.0 (286)	60.0 (154)	17.0 (98)	0
Vessels	3.0 (88)	4.0 (44)	1.0 (29)	0	3.0 (31)	0	0	0	29.0 (259)	0	2.0 (12)	3.0 (10)
Small buckets	7.0 (205)	5.0 (55)	0	0	3.0 (31)	0	13.0 (181)	22.0 (111)	5.0 (45)	6.0 (15)	9.0 (52)	0
Others (vases, tires, discarded, naturals, etc.)	14.0 (410)	1.0 (11)	4.0 (117)	3.0 (52)	0	0	2.0 (28)	0	0	0	0	0

Data collected in the treatment and control groups. It illustrates the overview of the study sites, as well as entomological indices and the relative contributions of specific containers in all 20 clusters and only in the 8 clusters completed until follow-up 2.

### Effectiveness of the first and second interventions: impact on entomological indices

The first intervention with LLIN curtains alone (with incomplete coverage of holes in the walls) (Figure 2) showed a reduction in entomological indices in intervention clusters compared to controls); the differences were statistically significant for BI only (p=0) (Table 3). BI fell from 14 to 6 (57%) in the intervention group and from 8 to 5 (38%) in the control group. PPI increased in 17% of the intervention houses but fell by 22% in control houses. The additional intervention with LLIN covers for water containers showed a significant reduction in vector densities measured through the PPI (p=0.01) as shown in Table 3. In the intervention group, the PPI showed a decline of 71% (from 0.75 at baseline to 0.22 at second follow up), compared with 25% (from 0.40 to 0.30) in the control group (Table 2 and Table 3).

**Table 3.** Differences in differences between study groups calculated as pupae per person index (PPI)^a^, Breteau index (BI) and container index (CI) from baseline to 9 weeks and to 29 weeks follow-ups^b^

		Mean change in intervention clusters	Mean change in control clusters	Dif of dif (95% CI)^c^	Wilcoxon p-value	t student p-value
PPI	After first intervention (baseline to 1st follow-up)	0.129 (from 0.75 to 0.88)	−0.096 (from 0.4 to 0.31)	0.225 (−0.125 to 0.49)	NS	0
	After second intervention (baseline to 2nd follow-up)	−0.501 (from 0.72 to 0.22)	−0.055 (from 0.36 to 0.3)	−0.446 (−0.49 to −0.40)	0.01	0
	1st vs 2nd follow-up	−0.936 (from 1.15 to 0.22)	0.011 from 0.29 to 0.3)	−0.947 (−0.99 to −0.89)	0.01	0
BI	After first intervention (baseline to 1st follow-up)	−0.079 (from 0.14 to 0.06)	−0.031 (from 0.08 to 0.05)	−0.049 (−0.051 to −0.046)	0	0
	After second intervention (baseline to 2nd follow-up)	−0.078 (from 0.12 to 0.06)	−0.05 (from 0.08 to 0.03)	−0.029 (−0.084 to 0.02)	NS	0
	1st vs 2nd follow-up	−0.038 (from 0.08 to 0.05)	−0.013 (from 0.05 to 0.03)	−0.025 (−0.029 to −0.02)	NS	0
CI	After first intervention (baseline to 1st follow-up)	−0.024 (from 0.042 to 0.018)	−0.016 (from 0.034 to 0.018)	−0.008 (−0.009 to −0.006)	NS	0
	After second intervention (baseline to 2nd follow-up)	−0.028 (from 0.047 to 0.02)	−0.02 (from 0.035 to 0.015)	−0.01 (−0.01 to −0.005)	NS	0
	1st vs 2nd follow-up	−0.005 (from 0.024 to 0.019)	−0.004 (from 0.019 to 0.015)	−0.001 (−0.002 to 0.001)	NS	NS

NS: not significant.

^a^ PPI median values: baseline with 20 clusters: intervention clusters: 0.7, control clusters: 0.33; diff 0.38; p value: 0.04 (Wilcoxon test); first follow-up with 20 clusters: intervention clusters: 0.72, control clusters: 0.18, diff 0.54, p value 0.01 (Wilcoxon test); second follow-up with 8 clusters: intervention clusters: 0.24, control clusters: 0.09, diff 0.15, p value NS (Wilcoxon test); baseline with 8 clusters: intervention clusters: 0.72, control clusters: 0.22; first follow up with 8 clusters: intervention clusters: 1.15, control clusters: 0.29.

^b^ Mixed model analysis showed no significant variation of PPI at the cluster level.

^c^ 95% CIs.

The majority of containers that produced >78% of pupae were containers >200 L; after the intervention with LLIN covers the pupae productivity decreased from 970 to 388 (60%) and in the control group from 394 to 339 (16%). The other container types were responsible for <12% pupal production, especially small buckets (Table 2).

### Coverage and satisfaction towards intervention

Out of the 947 eligible households, 97.4% (922) households received 3138 window and door curtains (2216 for windows and 922 for doors). After 9 weeks, 74.1% (577/779 of houses with available data) of the households were still using at least one curtain; 74.9% (432/577) of residents reported a willingness to pay and 89.6% (517/577) of householders would recommend them to friends and neighbours. A total of 78.0% (450/577) of the residents reported good quality of the material and 32.1% (185/577) rated the quality of the curtains 5 (excellent) on a 1 to 5 scale. After two follow-ups (29 weeks), the percentage of use decreased to 45.2% (417/922) because the monitoring period coincided with the Christmas holidays and new year seasons, when 34.8% (77/221) inhabitants removed curtains in order to decorate and paint their houses; 11.7% (26/221) washed them and 6.3% (14/221) traveled or migrated to other places.

Out of 385 eligible households, 78.7% (303) households received 354 water container covers for 303 rectangular and 51 circular containers. After using them, 60.1% (182/303) of residents reported a willingness to pay for the covers, 83.2% (252/303) would recommend them to friends and neighbours, and 89.8% (272/303) would be happy to receive them again if they were free; 26.4% (80/303) reported less use of do-it-yourself insecticide sprays and indicated that the median cost of the sprays was US$8.

The 10 FGD showed that participants were impressed by the number and diversity of dead insects (mosquitoes, cockroaches, domestic flies, grasshoppers and other domestic pests) below curtains; they would all recommend the interventions to others. Mild cutaneous rash was reported by the seamstresses who manipulated the LLIN material without using gloves or other protective gear (2/10 tailors). This effect was also reported by 2.7% of residents (21/779) who received curtains.

### Costs

The cost per household reached with the first intervention was US$28.8 and with the second intervention US$19.2. Cost per house: personnel (US$2.89), transport (US$0.48), additionally costs associated to community mobilisation (US$1.52) are relatively small compared to the LLINs and installation. Personnel were the main driver of the costs of the routine activities accounting for 57% of the total costs while larvicide represented less than 2% of the total costs. With 20 845 houses in the municipality, routine activities costs nearly US$4.9 per house. Options for cost savings were identified.

## Discussion

### Effectiveness

To our knowledge, this is the first report of a cluster randomised control trial to address LLINs for dengue vector control in Colombia. The first intervention with LLIN curtains in windows and doors achieved only an incomplete coverage of other household holes. Nevertheless, a significant reduction in BI in the intervention clusters was encountered when compared to control clusters; differences were not significant for PPI as a proxy for adult vector densities. This could be explained by changes in oviposition site selection.^26^ Curtains may have produced changes in a small fraction of the adult mosquito population as a physical barrier or as a consequence of the demonstrated repellent and deterrent effects of pyrethroids.^27^ As incomplete coverage was achieved many mosquitoes may have escaped and successfully oviposture in the most productive container (low water containers) as reflected in the PPI.

The combination of LLIN curtains with targeted interventions on the main productive water containers showed a significant reduction in vector densities when measured through PPI. If a mosquito was able to escape the effects of the curtains, they would still encounter difficulties in laying eggs and, even if successful, the emerging mosquitoes would be killed by treated container covers. This underlines the importance of combined vector control strategies. According to the computer simulation models developed by Focks et al.^28^ the pupal density of PPI=0.26 in intervention clusters (compared to a PPI=0.36 in control clusters) was below the threshold of epidemic dengue transmission when considering a temperature of 30°C and a seroprevalence of 66%.

Our result is similar to a study from Guatemala^6^ where LLIN window curtains alone resulted in a non-significant reduction in dengue vector density but, in combination with a targeted intervention in productive container types, resulted in a significant decrease. Kroeger et al.^4^ found in Mexico and Venezuela—where windows could be completely covered with insecticide-treated curtains—that these significantly reduced vector densities in intervention clusters, and that there was a clear spillover effect in the contact areas of intervention and control clusters.

In our study, there were several factors potentially contributing to a reduced effectiveness of the intervention: the continued actions of the vector control programme in both control and intervention clusters; the neglect of many unprotected holes in the walls; peoples' habit of ‘folding’ the curtains during the heat of the day; and the influx of *Aedes* from the unprotected surrounding areas of the intervention clusters

### Feasibility

Our study did show that the intervention package was effective in reducing PPI, but the feasibility of any control programme does require additional elements such as people's, satisfaction with the control services, and affordable costs.

Acceptance was achieved by the use of participatory strategies reported by García-Betancourt et al.^22^ Partnerships were established among local authorities, vector control services and the community. Satisfaction towards intervention was enhanced as people observed the dead insects below the curtains, which convinced them of its efficacy. This is similar to the findings reported in Haití,^17^ Venezuela^4^ and the Guatemala study.^6^ Yet in spite of people's positive perceptions, after some time they forgot about the benefits of the interventions and stopped using them: this means that there is a need for continued encouragement and monitoring of the intervention programme to sustain LLIN coverage which has a coverage-dependent effect on vector densities, as reported by Vanlerberghe et al.^10^

Based on the preliminary results of the intervention, vector control staff and local policy makers were very much in favour of continuing the novel vector control tool. National decision makers expressed an interest in financing the extension of the study to larger parts of the city and measuring whether reductions in dengue incidence could be achieved.

As a general rule, community-based interventions require more time and resources than conventional institution-based interventions because of longer socialisation and negotiation processes that are needed to achieve social participation and to respond to community expectations. The median costs of the intervention package in Girardot were US$48 per household: considerable if compared with similar interventions implemented in other countries. Rizzo et al.^6^ reported a median cost of US$5.31 per household for the same intervention package in Guatemala, excluding the costs of the netting materials that were donated. Baly et al.^29^ estimated median costs of US$8.84 and US$6.68 per household cover with LLINs in Venezuela and Thailand, respectively, also excluding the costs of the LLIN materials. However, the cost savings by families where dengue cases have been averted are also substantial. A recent study showed that households have out-of-pocket expenditure for dengue prevention of US$13.27 but an indirect cost of US$197.10 for dengue outpatients.^3^ Thus, the median costs of the intervention in our study are high, but when compared to local out-of-pocket expenditures or indirect costs, the investment in the described intervention package seems to be warranted.

### Limitations

Our impact evaluation is based on a short observation period; we do not know for how long after a 5 and 12 month observation period the effect will be sustainable. The efficacy, along with other vector control tools, will depend on multiple factors such as appropriate actions of social mobilisation to achieve long lasting behaviours, positive perceptions, perceived effectiveness, durability of materials used for interventions, coverage attained and local contextual and environmental conditions. However, the preliminary results are encouraging for policy makers.

Baseline and follow-up surveys took longer than initially planned, spreading over 3 months, as some houses were only occupied during holiday seasons, therefore field workers spent extra time revisiting empty houses to ensure participation. This could have biased our results as infestation rates can change according to season. However, our previous study^15^ demonstrated that pupal productivity is independent of rainfall, as no significant differences were found between seasons. Indoor low water container covers alone produce more than 70% of *Aedes* pupae during the wet and dry season and there are very few productive water containers outdoors.

Working with local seamstresses and SMEs that produced the aluminium frames for covering water tanks was beneficial. However, in the case of the container covers, SMEs were not prepared for such a high workload and resource utilisation. Therefore, frequent interruptions between measuring water container size and installation of covers were the rule.

### Conclusions

The results obtained in our study indicate that the intervention package can reduce dengue vector density. However, this success, as well as all vector control tools, will depend on multiple factors. Successful and adequate use of the intervention packages should be enhanced through appropriate social mobilisation to achieve long-lasting behavioural change.
